# Extensive Evaluation of a Method for Quantitative Measurement of Aflatoxins B1 and M1 in Animal Urine Using High-Performance Liquid Chromatography with Fluorescence Detection

**DOI:** 10.1093/jaoacint/qsad034

**Published:** 2023-03-13

**Authors:** Xiangwei Du, Dwayne E Schrunk, Paula M Imerman, John Tahara, Andriy Tkachenko, Jake Guag, Renate Reimschuessel, Wilson K Rumbeiha

**Affiliations:** Veterinary Medical Diagnostic Laboratory, Department of Biomedical Sciences, University of Missouri, 901 East Campus Loop, Columbia, MO 65211, USA; Veterinary Diagnostic Laboratory, Department of Veterinary Diagnostic and Production Animal Medicine, Iowa State University, 1850 Christensen Dr, Ames, IA 50011, USA; Veterinary Diagnostic Laboratory, Department of Veterinary Diagnostic and Production Animal Medicine, Iowa State University, 1850 Christensen Dr, Ames, IA 50011, USA; California Animal Health and Food Safety Laboratory System, Toxicology Laboratory, University of California, Davis, CA 95616, USA; United States Food and Drug Administration, Center for Veterinary Medicine, 8401 Muirkirk Rd, Laurel, MD 20708, USA; United States Food and Drug Administration, Center for Veterinary Medicine, 8401 Muirkirk Rd, Laurel, MD 20708, USA; United States Food and Drug Administration, Center for Veterinary Medicine, 8401 Muirkirk Rd, Laurel, MD 20708, USA; Department of Molecular Biosciences, University of California, Davis, CA 95616, USA

## Abstract

**Background:**

Aflatoxins (AFs) are common feed contaminants and are one of the common causes of toxin-related pet food poisoning and recalls.

**Objective:**

Currently, there are no validated methods for the detection and quantitation of AFs in biological matrices to diagnose AF exposure in live animals. Following a successful intra-laboratory method development to quantify AFB_1_ and AFM_1_ in animal urine by HPLC with fluorescence detection (HPLC–FLD), the present study was conducted to extensively evaluate the method performance in an unbiased manner using blinded samples.

**Methods:**

The evaluation included two stages. First, the performance was verified in the method-originating laboratory in a single-laboratory blinded method test (BMT-S) trial followed by a multi-laboratory blinded method test (BMT-M) trial.

**Results:**

In both trials, accuracy, repeatability, and reproducibility were satisfactory confirming the relatively good ruggedness and robustness of the method and ensuring that it will perform as expected if used by other laboratories in the future.

**Conclusions:**

We extensively evaluated the performance of a quantitative method to detect AFB1 and AFM1 in animal urine by HPLC-FLD by two different laboratories in two separate BMT-S and BMT-M trials. Both BMT results demonstrated the satisfactory accuracy and precision of the method. It is now available to be adopted by other diagnostic laboratories for purposes of diagnosing AF intoxication in animals.

**Highlights:**

A simple urine-based diagnostic test method using HPLC–FLD that originated in a single laboratory now has passed a multi-laboratory evaluation and is now available to be shared with other diagnostic laboratories for purposes of diagnosing AF intoxication in animals so better treatment can be rendered.

AFs are a family of potent mycotoxins produced by several species of fungi (1,2), which can be found in a wide variety of feed and food products (3). These fungi produce toxins, which cause acute and/or chronic liver disease in animals. Aflatoxins (AFs) B_1_, B_2_, G_1_, and G_2_ are the most common AFs (Figure 1), with AFB_1_ being the most potent AF with respect to toxicity and carcinogenesis (4, 5).

**Figure 1.**
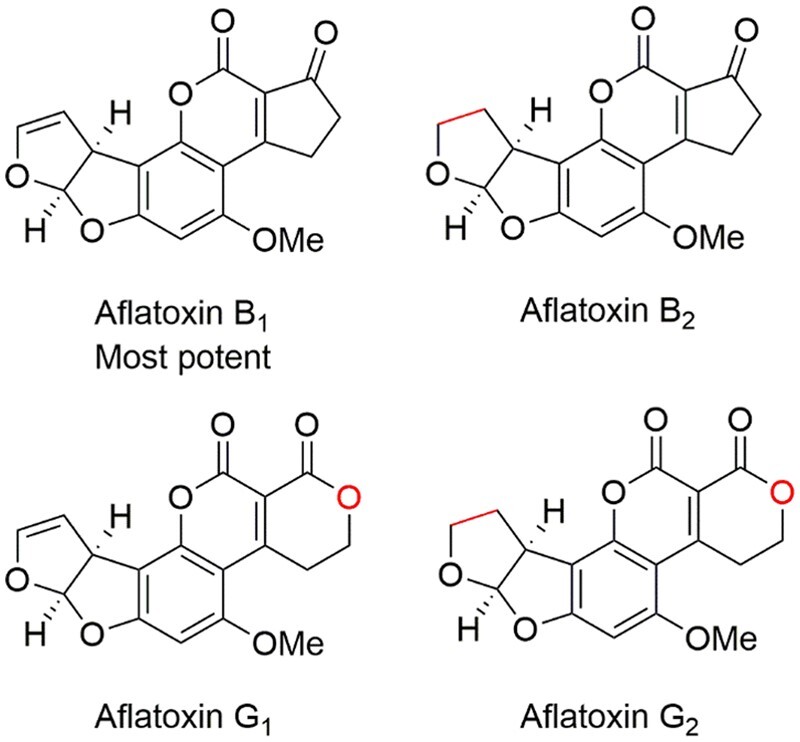
Structure differences of the four parent AFs.

After ingestion of AF-contaminated foodstuffs, AFB_1_ is metabolized by liver enzymes into several metabolites, including AFM_1_, AFP_1__*,*_ and AFQ_1_ (6). These metabolites cause multiple toxic effects including hepatotoxicity, immunosuppression (7), mutagenesis (8), teratogenesis (9), impaired reproduction, suppressed milk production (10), and carcinogenesis in animals (11–14). AFM_1_ is a major metabolite of AFB_1_ in animals and humans and is generated via cytochrome P450 metabolism. In terms of toxicity, AFM_1_ is as potent as AFB_1_ (15, 16). Currently, there is a gap in the diagnosis of AF exposure in animals, as there are no validated test methods for definitive confirmation of exposure or diagnosis of aflatoxicosis in live animals.

Recently, we developed a method for the quantitation of AFB_1_ and AFM_1_ in animal urine (17), which was validated in the method-originating laboratory using unblinded samples. The method employs a commercially available immunoaffinity column for clean-up, HPLC with fluorescence detection, and pre-column derivatization to increase sensitivity. The method is highly selective: recovery is > 81%. Also, the method which has an LOQ of 0.77–4.46 pg was found to have high accuracy, repeatability, and ruggedness (17). To ensure that the newly developed method will perform as expected in other laboratories, herein, we extensively evaluated the method performance in two laboratories in two separate blinded method test (BMT) trials. In both trials, participants were required to analyze unbiased (i.e., blinded) samples prepared by an independent laboratory. For the single-laboratory blinded method test (BMT-S) trial, the analysis was performed by the method-originating laboratory only. For the multi-laboratory blinded method test (BMT-M), the analysis was completed by the method-originating laboratory together with an additional collaborating laboratory.

## Experimental

### General Experimental Design of the Collaborative Evaluation

The in-house method validation work, which was completed (17) at Iowa State University (ISU) according to United States Food and Drug Administration (FDA) guidelines (18) and reviewed by the Veterinary Laboratory Investigation and Response Network (Vet-LIRN), FDA, was the pre-requisite for BMT-S. After the BMT-S data were found to be satisfactory, a further evaluation of the method via a multi-laboratory blinded method test (BMT-M) was conducted by the organizers at Level 3 (i.e., requiring at least two collaborating laboratories) according to FDA guidelines in place at the time of the study (18). Both BMT-S and BMT-M were based on the same principles as described previously (19) to fully blind the participating laboratories regarding the composition of BMT samples thereby eliminating the participant’s conscious and unconscious bias during sample analysis and data assessment (20). The participating laboratories were not aware of any of the following regarding BMT samples: (1) the number of analyte levels used; (2) the number of replicates used for each analyte level, and (3) the analyte concentrations used in each analyte level.

During the analysis of BMT-M samples, both participating laboratories were required to complete analyst worksheets (AW), which were designed by the organizer (i.e., Vet-LIRN) based on the method’s standard operation procedures (SOP), with dual purposes: (1) to capture details of all steps performed by each analyst including details on consumables and equipment used, and (2) to assess multiple performance parameters of the method. All results and raw data (e.g., completed AW, integrated chromatograms, and their peak intensities) were submitted by the two participating laboratories directly to organizers for preliminary evaluation. Submitted results were statistically summarized according to FDA (18) and AOAC INTERNATIONAL (21–23) recommendations by the ISU Veterinary Diagnostic Laboratory (VDL) in conjunction with organizers. From 14 to 18% of samples were designated “mystery” samples with one replicate only, which were prepared at concentrations different from those which were used for replicated samples (19). Although all samples are unknown, because there is only one replicate, “mystery” samples eliminated the possibility that a test result obtained on one sample would influence a test result on another sample affecting the estimate of the method’s accuracy and precision. Their purpose was to minimize analyst bias toward possible clustering of similar results for replicated samples. Procedures that exclude one-replicated mystery samples facilitate the identification of outliers by participants even without knowing the exact concentration used in replicated samples. Mystery samples were not used for evaluation of the method performance but for discussion purposes.

### Chemicals and Standards

For both BMT trials, the organizer purchased fresh AFB_1_ and AFM_1_ standards from Sigma-Aldrich (St. Louis, MO, USA), prepared spiking standard solutions (200 ng/mL for each AF in methanol), and fortified the blank urine at various levels to prepare unknown (i.e., blinded) samples. The spiking standard solutions were delivered to participants for preparation of the calibration curves in urine along with the negative control and unknowns. The quantitative ranges of calibration curves were 0.3–15.0 ng/mL for AFB_1_ (0.3, 1.0, 2.0, 4.0, 8.0, and 15.0 parts per billion [ppb]) and 0.5–15.0 ng/mL for AFM_1_ (0.5, 1.0, 2.0, 4.0, 8.0, and 15.0 ppb). The two participating laboratories of the BMT-M were also provided with clean-up immunoaffinity columns (IACs) prepared in the method-originating laboratory.

Participating laboratory-1 used the following materials and reagents: HPLC grade methanol (MeOH) and acetonitrile (ACN), A.C.S. reagent grade potassium phosphate monobasic, potassium chloride, sodium phosphate dibasic, sodium chloride, trifluoroacetic acid (TFA), and glacial acetic acid were obtained from Fisher Scientific (Waltham, MA, USA). Phosphate-buffered saline (PBS) 1× solutions were made from potassium phosphate monobasic, potassium chloride, sodium phosphate dibasic, and sodium chloride with pH between 7.2 and 7.6.

AFLAPREP IACs were purchased from R-Biopharm AG (Washington, MO, USA). All aqueous solutions were prepared using 18.2 MΩ cm deionized water (Aries Filter Network, West Berlin, NJ, USA). Participating laboratory-2 used materials and reagents, which were equivalent to those used in laboratory-1 but from different sources allowing organizers to test ruggedness/robustness of the method in BMT-M.

### Samples

**Figure 2.**
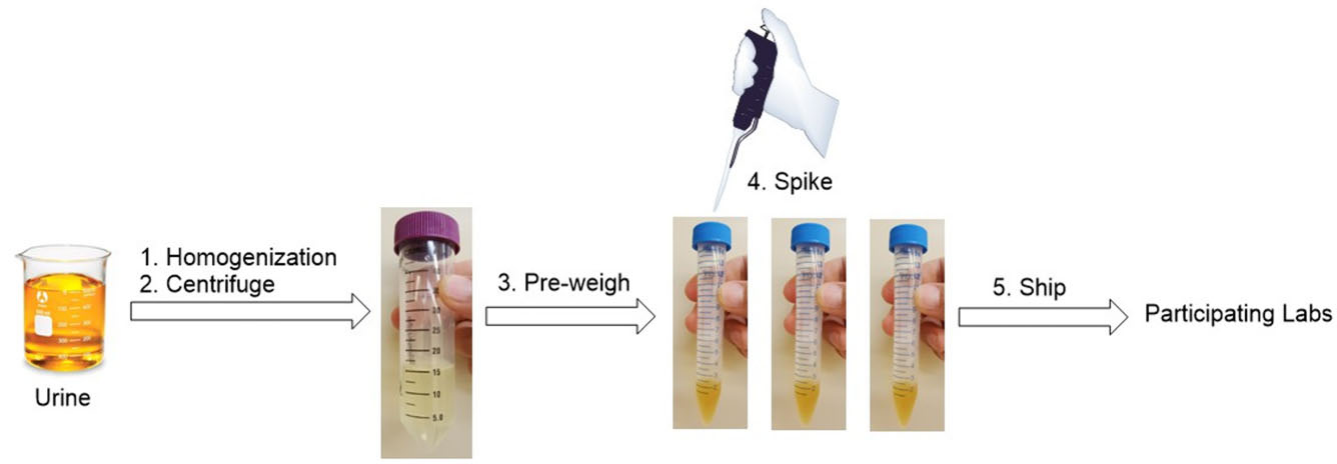
Preparation of fortified urine samples for BMT-S and BMT-M.

Canine urine was collected from adult greyhound racing dogs submitted to the ISU VDL according to the institution internal procedures. Urine samples that were negative for any illicit drugs were pooled. This pooled urine sample was then mixed by stirring for 10 min, centrifuged at 3040 × *g* for 10 min in 50 mL centrifuge tubes to remove particulate matter, tested to ensure it was negative for AFs, and stored at −80°C before use. The urine was shipped to organizers on dry ice where it was defrosted, centrifuged at 2500 × g for 15 min at 25°C to remove precipitates, aliquoted (2.0 ± 0.1 mL) into 15 mL polypropylene tubes, and fortified with AFB_1_ and AFM_1_ working solutions as illustrated in Figure 2. The fortified concentration level of AFB_1_ and AFM_1_ is based on the method LOQ (0.3 ng/mL for AFB_1_ and 0.5  ng/mL for AFM_1_). Du et al summarized clinically relevant urinary AF concentrations of AFB_1_ and AFM_1_ in pigs, steers, dogs, and rodents, and overall these were >0.5 ng/mL for AFB_1_ and >5 ng/mL for AFM_1_ (17).

### Instrumental Analysis

**Figure 3.**
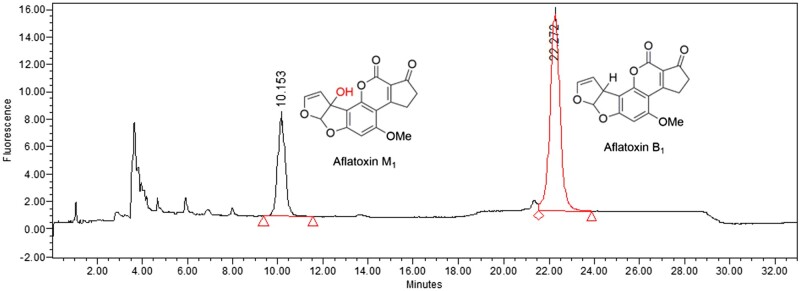
Representative HPLC chromatogram of AFB_1_ and AFM_1_ in urine at 2 ng/mL.

All measurements were performed on a HPLC instrument equipped with a fluorescence detector. A Phenomenex (Torrance, CA) Kinetex Biphenyl (2.6 μm, 100 mm × 4.6 mm) HPLC column and an Agilent (Santa Clara, CA) Pursuit XRs 3 C18 (2.0 mm) guard column were used. The mobile phase A is deionized water, while mobile phase B is acetonitrile with 1.0 mL/min flow rate and 20 μL injection volume at room temperature. The details of the gradient profile are shown in Table 1. The excitation and emission wavelengths for the fluorescence detector were 360 and 440 nm, respectively. A representative HPLC chromatogram of AFB_1_ and AFM_1_ in urine is shown in Figure 3.

**Table 1.** HPLC gradients for separating AFB_1_ and AFM_1_

Time, min	Mobile phase A: water, %	Mobile phase B: acetonitrile, %
0–1	100	0
1–2	84	16
2–16	84	16
16–17	80	20
17–27	80	20
27–28	100	0
28–33	100	0

### Stability

The stability of AFB_1_ and AFM_1_ in urine had been investigated during the in-house validation (17) by the method originators prior to both BMTs. Potential analyte stability issues were also minimized by the organizers as follows: (1) participants were required to analyze the samples within 15 business days after receipt and (2) participants were required to prepare calibration curves using the same AFB_1_ and AFM_1_ spiking standard solutions used by organizers to prepare unknown BMT samples and provided to participants. An archived set of samples was also prepared and stored by organizers for possible follow-up in case any stability questions arose.

### Sample Extraction and Clean-Up

Samples were processed by all participants in the BMT-S and BMT-M according to our previously published work (17). Briefly, 4.0 mL methanol–water (80 + 20, v/v) was added to each urine sample (2 mL) and mixed on a vortex mixer at maximum speed for 10 min using a Multi-Tube Vortexer (Fisher Scientific). Subsequently, 2.0 mL aliquots were transferred into 50 mL screw-cap polypropylene tubes, mixed with 14 mL 1× PBS solutions, and mixed on a vortex mixer at maximum speed for 5 s. The solutions were then loaded onto IACs (stored at 5°C and pre-adjusted to room temperature) and passed through the columns by gravity. IACs were then washed with 20 mL 1× PBS solutions by vacuum at a flow rate of 0.25–0.5 mL/min. The solutions were discarded, and the residual AFs were eluted from the IACs with 1.0 mL methanol followed by 1.0 mL water at a flow rate of one drop/s and collected in the same vial for each sample. IACs were flushed with nitrogen 3–4 times to eluate all eluents. The eluents were concentrated to dryness under a stream of nitrogen. The dried extracts were then subjected to a derivatization procedure before HPLC analysis based on optimized protocols for each laboratory (17). Briefly, the extracts were reconstituted in 400 µL water–TFA–glacial acetic acid (35 + 10 + 5, v/v/v), mixed on a vortex mixer for 10 s, and heated at 65°C in a heating dry bath for 15 min. Subsequently, the solutions were incubated for 20 h at room temperature before HPLC analysis.

### Evaluation of Data

The BMT-S and BMT-M participants were required to submit calculated results and raw data (e.g., completed AWs, integrated chromatograms, and their peak intensities) directly to organizers. Intra- and inter-laboratory accuracy and precision were the criteria used to evaluate method performance. To pass the acceptance criteria, according to the FDA guidelines, accuracy should fall within the 40–120% range for concentrations below 1 ng/mL and between 60 and 115% for concentrations between 1 and 10 ng/mL (18, 23). The RSD(r) and Horwitz Ratio (HorRat(r)) values were used for the evaluation of the intra-laboratory precision (i.e., repeatability, r), while RSD(R) and HorRat(R) values were used for inter-laboratory precision (i.e., reproducibility, R) according to FDA (18) and AOAC recommendations (23). To be acceptable, HorRat(r) values should fall within the 0.3–1.3 range, while the recommended acceptable HorRat(R) values should fall within the 0.5–2.0 range. Lower values within the acceptable range indicate excellent method performance (21–24). RSD(R) matched expectations too.

## Results and Discussion

### Single-Laboratory Blinded Method Test (BMT-S)

Twenty-two unknown samples were randomized by the organizer and analyzed by participating laboratory-1 on two separate days (11/day) to evaluate inter-day accuracy and precision of the method within the same laboratory. As shown in Table 2, the intra-laboratory accuracy obtained for blinded samples was 89–94% for AFB_1_ and 81–93% for AFM_1_ based on calculated average concentrations. The HorRat(r) values were 0.31–0.45 for AFB_1_ and 0.22–0.53 for AFM_1_, respectively. In addition, the correlation coefficient (*r*^2^) of calibration curves were >0.999 for AFB_1_ and >0.999 for AFM_1_. Back-calculated accuracy for all six calibrators was within ±20% of the nominal concentration (data are not shown) according to FDA recommendations (25). The intra-laboratory precision for both AFB_1_ and AFM_1_ was within the expected ranges indicating satisfactory repeatability of the method. The HorRat(r) values obtained were within or below the normally expected range of 0.3–1.3 (Table 2). RSD(r) values were within expected ranges too.

**Table 2.** Summary of results reported by laboratory-1 in BMT-S

Fortified level, ng/mL		Concentration found, ng/mL	Accuracy, %	Expected RSD(r), %	Found RSD(r), %	HorRat(r)
AFB_1_	0	ND^a^, ND	NA^b^	NA	NA	NA
	0.6^c^	0.57	95	NA	NA	NA
	1.1	0.80, 1.02, 0.95, 1.09, 0.98, 0.98, 1.05 (0.98)^d^	89	22	9.5	0.43
	4.6	4.22, 4.51, 3.88, 3.92, 4.20, 4.21 (4.16)^d^	90	18	5.5	0.31
	9.0^c^	8.10	90	NA	NA	NA
	10.0	9.55, 10.61, 9.11, 9.18, 8.64, 9.58 (9.45)	94	16	7.1	0.45
	11.0^c^	10.88	99	NA	NA	NA
AFM_1_	0.8^c^	0.75	94	NA	NA	NA
	1.0	0.89, 0.92, 0.88, 1.04, 0.98, 0.89 (0.93)^d^	93	23	9.8	0.44
	4.5	4.62, 4.32, 3.76, 4.14, 3.65, 3.79 (4.05)^d^	90	18	9.4	0.52
	9.0^c^	7.70	86	NA	NA	NA
	10.0	7.62, 8.59 (8.11)	81	16	8.4	0.53
	12.0	11.16, 10.29, 10.93, 10.42, 11.12, 10.96 (10.81)^d^	90	16	3.4	0.22

a NA = Not applicable.

b ND = Not detected.

c Calculated average concentration.

d Designated “mystery” samples (see the text for details).

### Multi-Laboratory Blinded Method Test (BMT-M)

Each of the two participating laboratories analyzed 22 randomized samples on two separate days (11/day) to evaluate inter-day accuracy and repeatability (i.e., precision within a laboratory) of the method within each laboratory. The average concentration, accuracy, and intra-laboratory precision (e.g., RSD(r) and HorRat(r)) values were calculated for each level. The results from participating laboratory-1 are shown in Table 3: the accuracy ranged from 93–96% for AFB_1_ and 97–105% for AFM_1_. The HorRat(r) values ranged from 0.42–0.82 for AFB_1_ and 0.33–0.56 for AFM_1_, respectively. The correlation coefficients (*r*^2^) of calibration curves (data are not shown) were >0.99 for AFB_1_ and >0.99 for AFM_1_. The back-calculated accuracy (25) for at least five of the six calibrators was within ±20% of the nominal concentration (data are not shown).

**Table 3.** A summary of results^c^ reported by laboratory-1 in BMT-M

Fortified level, ng/mL		Concentration found, ng/mL	Accuracy, %	Expected RSD(r), %	Found RSD(r), %	HorRat(r)
AFB_1_	0	ND, ND	NA	NA	NA	NA
	0.6^d^	0.50	83	NA	NA	NA
	1.1	1.04, 1.31, 1.28, 0.94, 0.80, 0.98, 0.93 (1.04)^a^	95	22	18	0.82
	4.7	4.20, 5.00, 4.91, 3.84, 4.66 4.54 (4.53)^a^	96	18	10	0.56
	9.0^d^	8.63	96	NA	NA	NA
	10.0^d^	9.71	97	NA	NA	NA
	11.0	4.51^b^, 9.18, 9.97, 10.25, 10.59, 10.97 (10.19)^a^	93	16	6.7	0.42
AFM_1_	0.8^d^	0.81	102	NA	NA	NA
	1.0	1.10, 1.17, 1.05, 0.86, 1.00, 1.07, 1.13, (1.06)^a^	105	23	10	0.43
	4.6	3.58, 4.71, 4.47, 4.59, 4.63, 4.73 (4.45)^a^	97	18	10	0.56
	9.0^d^	9.77	109	NA	NA	NA
	10.0^d^	9.89	99	NA	NA	NA
	12.0^d^	12.55	105	NA	NA	NA
	13.0	13.13, 12.64, 11.61, 12.38, 13.27 (12.61)^a^	97	15	5	0.33

a Calculated average concentration.

b The value was considered as an outlier and excluded from calculations. NA = Not applicable. ND = Not detected.

c Twenty-two samples were randomized by organizer and analyzed by participating laboratory-1 on two separate days (11/day), which were 12 days apart.

d Designated “mystery” samples (see the text for details).

The results from participating laboratory-2 are shown in Table 4. The accuracy was 77–89% for AFB_1_ and 77–83% for AFM_1_. The HorRat(r) values were 0.39–0.82 for AFB_1_ and 0.44–0.65 for AFM_1_, respectively. RSD(r) values were within expected ranges too. The correlation coefficients (*r*^2^) of calibration curves (data are not shown) were >0.999 for AFB_1_ and AFM_1_. The back-calculated accuracy (25) for at least five of the six calibrators was within ±20% of the nominal concentration (data are not shown).

**Table 4.** A summary of results^b^ reported by laboratory-2 in BMT-M

Fortified level, ng/mL		Concentration found, ng/mL	Accuracy, %	Expected RSD(R), %	Found RSD(R), %	HorRat(r)
AFB_1_	0	ND, ND	NA	NA	NA	NA
	0.6^c^	0.46	77	NA	NA	NA
	1.1	1.03, 0.94, 1.00, 0.67, 0.86, 0.82, 0.64 (0.85)^a^	77	22	18	0.82
	4.7	4.40, 4.40, 4.12, 4.10, 3.61, 4.37 (4.17)^a^	89	18	7	0.39
	9.0^c^	7.83	87	NA	NA	NA
	10.0^c^	8.67	87	NA	NA	NA
	11.0	10.23, 8.64, 9.01, 8.67, 10.61, 11.54 (9.79)^a^	89	15	12	0.80
AFM_1_	0.8^c^	0.34	42	NA	NA	NA
	1.0	0.92, 0.96, 0.69, 0.67, 0.69, 0.73, 0.76 (0.77)^a^	77	23	15	0.65
	4.6	4.04, 4.04, 3.84, 4.02, 3.25, 3.64 (3.81)^a^	83	18	8	0.44
	9.0^c^	7.66	85	NA	NA	NA
	10.0^c^	8.78	88	NA	NA	NA
	12.0^c^	11.47	96	NA	NA	NA
	13.0	10.22, 11.65, 9.84, 11.57, 10.10 (10.68)^a^	82	15	8	0.53

a Calculated average concentration. NA = Not applicable. ND = Not detected.

b Twenty-two samples were randomized by organizer and analyzed by participating laboratory-2 on two separate days (11/day), which were six days apart.

c Designated “mystery” samples (see the text for details).

Results from both laboratories, except mystery samples, were combined to evaluate inter-laboratory accuracy and precision (Table 5). The accuracy was 86–92% for AFB_1_ and 90–91% for AFM_1_. The calculated inter-laboratory accuracy for AFB_1_ and AFM_1_ fell within the FDA guideline’s recommended ranges (40–120% for levels below 1.0 ng/mL and 60–115% for 1–10 ng/mL). The HorRat(R) values for levels with multiple replicates were 0.25–0.44 for AFB_1_ and 0.33–0.44 for AFM_1_, respectively, which met or exceeded expectations. RSD(R) values were within the expected ranges as well.

**Table 5.** A summary of combined results from two participating laboratories

Fortified level, ng/mL		Calculated average concentration, ng/mL	Accuracy, %	Expected RSD(R) %	Found RSD(R) %	HorRat(R)
AFB_1_	1.1	0.95	86	45	20	0.44
	4.7	4.35	92	36	9	0.25
	11.0	9.97	91	32	10	0.31
AFM_1_	1.0	0.91	91	45	20	0.44
	4.6	4.13	90	36	12	0.33
	13.0	11.64	90	31	11	0.35

## Conclusions

In this study, we extensively evaluated the performance of a quantitative method to detect AFB_1_ and AFM_1_ in animal urine by HPLC with fluorescence detection (HPLC–FLD). A relatively large number of samples (i.e., 68) were analyzed by two different laboratories in two separate trials. Both the BMT-S and BMT-M results demonstrated satisfactory accuracy and precision of the method. The method is also relatively rugged and robust because in each of the two BMTs each laboratory analyzed samples on two separate days. Moreover, laboratory-2 used equivalent but different equipment (e.g., HPLC–FLD system, centrifuge, solid-phase extraction apparatus, shaker, and pipettes), materials (e.g., tubes, filters, and tips), and reagents versus laboratory-1, and yet all results matched well between the two laboratories. In both BMTs, participating laboratories analyzed properly blinded (i.e., unbiased) samples, which were prepared in an independent laboratory. Such unbiased evaluation of the method’s performance provides a high degree of confidence that the method will perform as expected if used in other laboratories in the future.
